# Complete mitogenome of the invasive land flatworm *Parakontikia ventrolineata*, the second Geoplanidae (Platyhelminthes) to display an unusually long *cox2* gene

**DOI:** 10.1080/23802359.2020.1765709

**Published:** 2020-05-18

**Authors:** Romain Gastineau, Jean-Lou Justine

**Affiliations:** a Institute of Marine and Environmental Sciences, University of Szczecin, Szczecin, Poland; b Institut Systématique Évolution Biodiversité (ISYEB), Muséum National d’Histoire Naturelle, CNRS, Sorbonne Université, EPHE, Université des Antilles, Paris, France

**Keywords:** Parakontikia ventrolineata, invasive, flatworm, Rhynchodeminae, *cox2*

## Abstract

We sequenced the complete mitogenome of the invasive flatworm *Parakontikia ventrolineata* (Platyhelminthes, order Tricladida, family Geoplanidae). The genome is 17,210 bp long, and displays common unusual characteristics shared with *Platydemus manokwari*, such as its colinearity, an overlap between *ND4L* and *ND4* genes and an unusually long *cox2* genes. Both *Parakontikia* and *Platydemus* are members of the subfamily Rhynchodeminae and their close relationships are supported by the maximum likelihood phylogeny inferred from the protein-coding genes.

*Parakontikia ventrolineata* (Dendy 1892) Winsor, 1991 is an invasive flatworm originating from Australia (Dendy 1892; Winsor 1991). The species is sometimes designated under the binomial *Kontikia ventrolineata* (Dendy 1892). It has been recorded in various parts of the world including the United States, Mexico, United Kingdom, Ireland, Spain and France (Alvarez-Presas et al. 2014; Sluys 2016). With its size comprised between 1 and 5 cm, it is small enough to enter inside damaged fruits, especially strawberries and apples, and as such is considered a nuisance by gardeners (Justine et al. 2014). *Parakontikia ventrolineata* has a generalist diet, including snails, slugs, and woodlice, and is also a scavenger on dead, crushed earthworms and snails (Winsor et al. 2004; Justine et al. 2014).

We sequenced the complete mitogenome of *P. ventrolineata*. The specimen used is part of a series of 10 specimens collected in Caen, France, on 26 March 2015 and deposited in the collections of the Muséum national d’Histoire naturelle, Paris, under number MNHN JL240 and where the rest of the specimen is kept. Paired end sequencing was conducted by the Beijing Genomics Institute (Shenzhen) on a DNBSEQ platform, resulting in a total of 60 million 100-bp reads. Assembly was performed using with SPAdes 3.14.0 (Bankevich et al. 2012) with a k-mer of 85. Genes were identified with the help of MITOS (Bernt et al. 2013).

The genome is 17210 bp long (GenBank accession number: MT081960) and it codes for 13 proteins, 2 rRNAs, and 21 tRNAs. It appears to be very similar to the recently sequenced mitogenome of *Platydemus manokwari* (GenBank: MT081580; Gastineau et al. 2020), an invasive flatworm (see Justine et al. 2014; 2015) also belonging to the subfamily Rhynchodeminae. Genomes are colinear including for the positions of tRNA, the open reading frames corresponding to the *ND4L* and *ND4* genes are overlapping by an identical lenght of 32 nucleotides and especially the *cox2* gene displays a similar overlength. The total length of the *cox2* gene is 1302 bp, close to the size observed in *P. manokwari* (1359 bp) and twice for example the 678 bp of the invasive flatworm *Bipalium kewense*’s *cox2* gene (GenBank: MK455837; Gastineau et al. 2019). This extension occurs between the same conserved protein domains as in *P. manokwari*.

A maximum likelihood phylogeny was inferred from concatenated gene-coding proteins with the protocol and dataset of Gastineau et al. (2020), using RAxML v.8.2.3 (Stamatakis 2014) and the MtArt substitution model (Abascal et al. 2007). The phylogeny strongly associates *P. ventrolineata* and *P. manokwari* in a single clade (Figure 1) and strictly discriminates them from the other Geoplanidae, *B. kewense* and *Obama nungara*, which are also invasive species (e.g. Justine et al. 2018, 2020). *Parakontikia* and *Platydemus* are both members of the subfamily Rhynchodeminae within the Geoplanidae. They were classified, however, in two different tribes within the Rhynchodeminae, i.e. the Rhynchodemini for *Platydemus* and the Caenoplanini for *Parakontikia* (Sluys et al. 2009). The family Geoplanidae includes four subfamilies, Microplaninae, Bipaliinae, Rhynchodeminae and Geoplaninae (Sluys et al. 2009); complete mitogenomes are now available for three of these, excluding the first one. The present findings of the shared characteristics of the *cox2*, *ND4L* and *ND4* genes suggest that these are common characteristics of members of the Rhynchodeminae, separating them from the other subfamilies. Our study emphasizes the interest of complete mitogenome for the understanding of phylogeny in the Geoplanidae.

**Figure 1.**
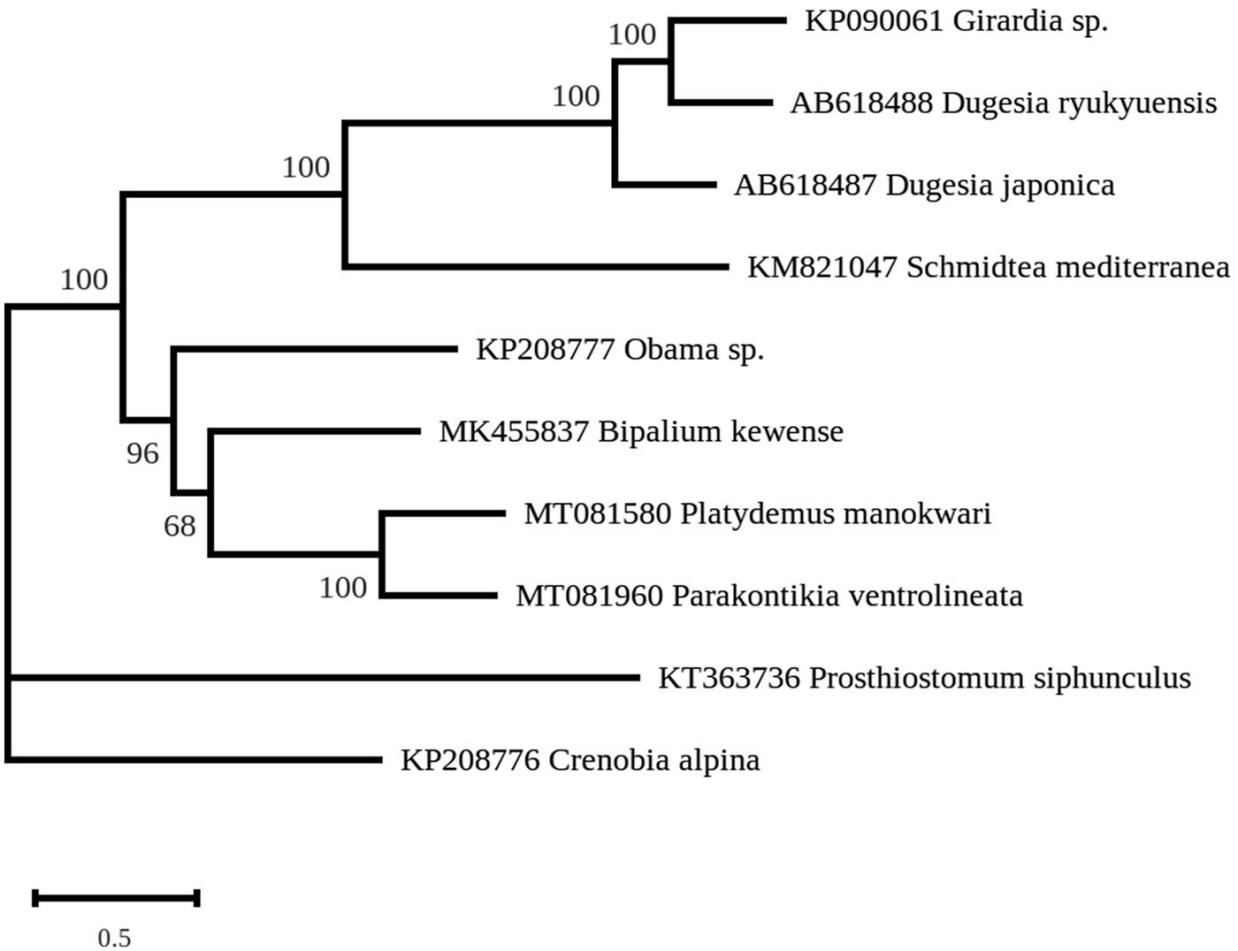
Maximum-likelihood tree obtained on concatenated amino-acid sequences of all mitochondrial protein coding genes from *Parakontikia ventrolineata* and other flatworms, using the MtArt model of evolution and after 100 bootstrap replications.

## Data Availability

The authors confirm that the data supporting the findings of this study are available at the following link: https://doi.org/10.5281/zenodo.3782525. The genome has also been deposited on GenBank with accession number MT081960.
